# Supplementary material for: Strategies for Efficient Expression of Heterologous Monosaccharide Transporters in Saccharomyces cerevisiae
Source: J Fungi (Basel). 2022 Jan 15;8(1):84. doi: 10.3390/jof8010084 (PMC8778384; doi:10.3390/jof8010084)
Supplement: Supplementary file 1 [file jof-08-00084-s001.zip › jof-1512031-supplementary.pdf]

Journal of Fungi

MDPI

Supplementary Material:

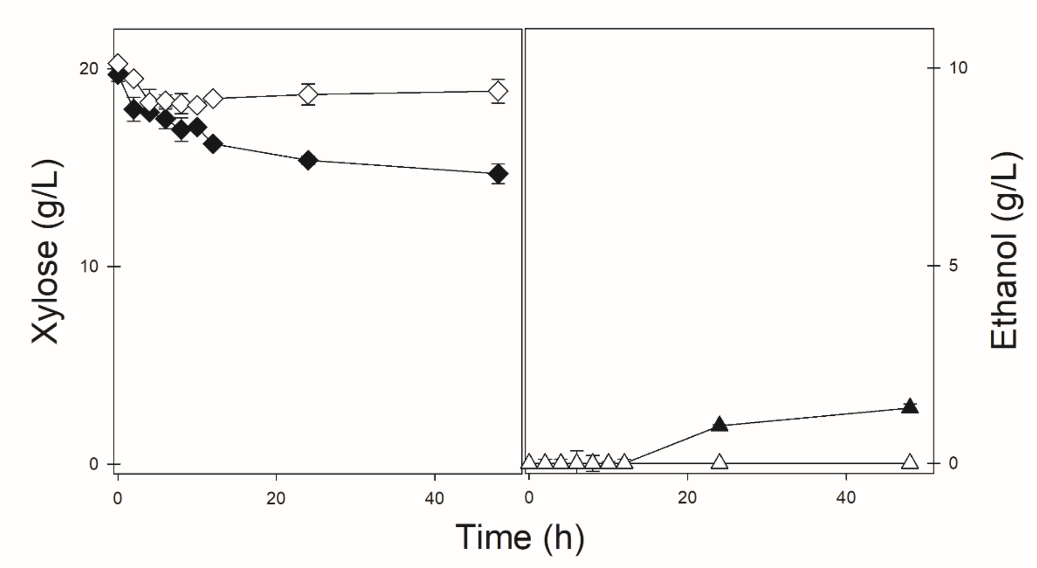

Supplementary Figure S1. Xylose fermentation under microaerobic conditions by the DLG-K1 strain transformed with the pGPD-*SaXUT1* (open symbols) or pGPD-*SaXUT1*ΔNC (black symbols) plasmids. The batch fermentation was performed with high cell concentrations (10 g DCW/L), at 30°C, and the concentration of xylose (diamonds) and ethanol (tringles) in the medium were determined at the indicated time points.

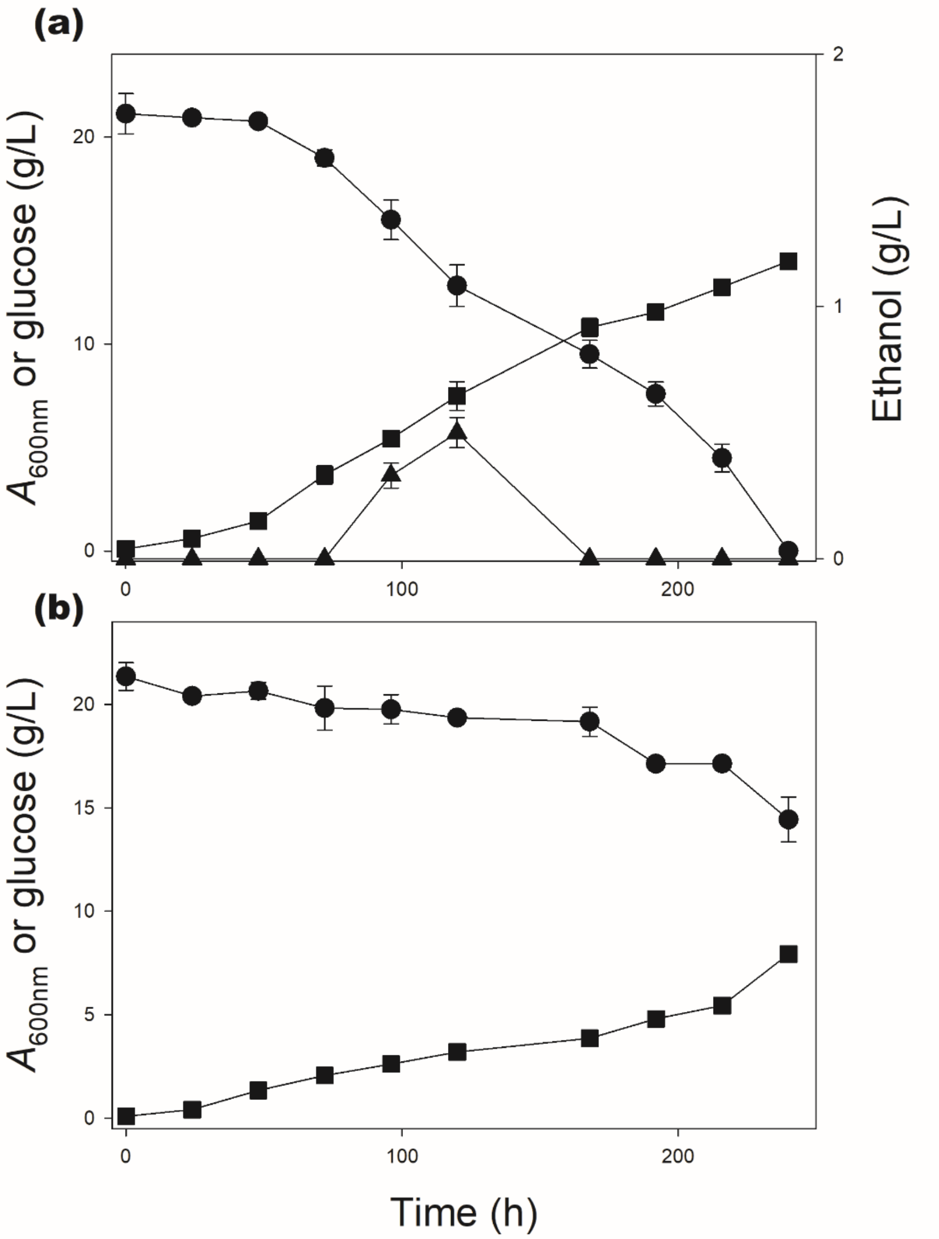

Supplementary Figure S2. Aerobic growth of strain DLG-K1ΔR3 transformed with plasmid pPGK-*SpXUT1* (**a**) or plasmid pGPD-*SaXUT1* (**b**) in O-YNB medium (lacking uracil) containing 2% glucose, at 30°C. At the indicated time points the cell growth (squares), glucose (circles), and ethanol (triangles) concentrations were determined. No ethanol was produced during glucose consumption by strain DLG-K1ΔR3 transformed with plasmid pGPD-*SaXUT1* (**b**).
